# Postoperative results and complications of fecal diversion for anorectal Crohn’s disease

**DOI:** 10.1007/s00595-022-02556-x

**Published:** 2022-07-22

**Authors:** Hirosuke Kuroki, Akira Sugita, Kazutaka Koganei, Kenji Tatsumi, Eiichi Nakao, Nao Obara

**Affiliations:** Department of Surgery for Inflammatory Bowel Disease, Yokohama Municipal Citizen’s Hospital, 1-1, Mitsuzawanishicho Kanagawa-ku, Yokohama City, 221-0855 Japan

**Keywords:** Crohn’s disease, Anorectal disease, Fecal diversion, Proctectomy

## Abstract

**Purpose:**

Fecal diversion is a less-invasive technique that can alleviate symptoms in patients with refractory anorectal Crohn’s disease. However, complications, including recurrence of residual anorectal Crohn’s disease, may develop. We aimed to evaluate the postoperative results and complications associated with fecal diversion in patients with refractory anorectal Crohn’s disease.

**Methods:**

We enrolled 1218 Crohn’s disease patients who underwent laparotomy at our institute. We retrospectively analyzed the clinical features of 174 patients who underwent fecal diversion for refractory anorectal Crohn’s disease, complications of the diverted colorectum, and the incidence and risk factors for proctectomy after fecal diversion.

**Results:**

After fecal diversion, 74% of patients showed improved symptoms. However, bowel continuity restoration was successful in four patients (2.2%), and anorectal Crohn’s disease recurred in all patients. Seventeen patients developed cancer with a poor prognosis. The rate of conversion to proctectomy after fecal diversion was 41.3%, and the risk factors included rectal involvement (*p* = 0.02), loop-type stoma (*p* < 0.01), and the absence of treatment with biologics after fecal diversion (*p* = 0.03).

**Conclusion:**

Fecal diversion for refractory anorectal Crohn’s disease can improve clinical symptoms. Patients with rectal involvement or loop-type stoma have a greater risk of requiring proctectomy following fecal diversion. The administration of biologic may decrease the rate of proctectomy.

## Introduction

The incidence of anorectal lesions in patients with Crohn’s disease (CD) is 20–80% [1–3]. A considerable number of such patients (20–49%) undergo surgical intervention, including diverting stoma or proctectomy [4–7]. Fecal diversion (FD) is less invasive than proctectomy and can alleviate the symptoms; however, many patients experience a worsening of clinical anorectal symptoms after stoma reversal [8]. The European Crohn’s and Colitis Organization (ECCO) stated that pelvic sepsis and its symptoms from complex perianal CD that is refractory to medical or surgical intervention can be controlled through sepsis drainage by a diverting stoma. Additionally, the ECCO stated that a diverting stoma may offer an alternative to extensive resection or proctectomy, allowing time for the acceptance of a permanent stoma due to scant evidence and a decreased rate of fistula healing [9]. However, a meta-analysis reported that the failure of temporary FD requiring proctectomy occurred in 41.6% of cases [8, 10–20]. Additionally, several authors have reported that the independent predictors of proctectomy after fecal diversion include age, the first incidence of anoperineal disease, and rectal involvement by CD [13, 20].

This retrospective single-institution study aimed to evaluate the postoperative results, complications, and rate of proctectomy in patients who undergo FD for refractory or severe anorectal CD.

## Materials and methods

### Patient selection

We included consecutive CD patients who underwent FD for refractory anorectal lesions between January 1999 and 2017. FD cases for other indications rather than anorectal lesions were not included in the analysis. The characteristics and the clinical course of CD patients were reviewed based on our institutional database and individual chart data.

The data set included sex, the age at the onset of CD, the extent of CD, the type of anorectal lesion (perianal fistula alone, anorectal stricture alone, rectal involvement defined as perirectal abscess or fistula, genital fistula, and perianal fistula with stricture), surgical techniques (i.e., abscess drainage or seton placement), site of stoma, type of initial stoma, duration from the diagnosis of CD to FD, continuous symptoms after FD, and the administration of biologics after FD.

This study was approved by the Ethical Advisory Committee of Yokohama Municipal Citizen's Hospital (21-05-06). The requirement for informed consent was waived because of the retrospective nature of the study.

### Perioperative management and surgical procedure

The standard treatments included 5-aminosalicylic acid (3000 mg/day), prednisolone, immuno-modulators (azathioprine or 6-mercaptopurine), and biologics (infliximab [5–10 mg/kg] or adalimumab) for CD-associated lesions without stricture or infection.

FD, which includes the creation of a loop stoma without resection of the diseased colon and rectum or Hartmann’s procedure, is one of the standard surgical procedures for patients with CD with severe colorectal disease. Many Japanese patients tend to select FD to preserve the anus, considering the possibility of subsequent stoma reversal. Younger patients tend to avoid proctectomy with abdominoperineal excision (APE) or total proctocolectomy (TPC) with ileostomy because of the possibility of sexual and urinary dysfunction following these procedures. Proctectomy was defined as APE or TPC. Most loop stomas were constructed in the ileum, except for cases involving patients with a short residual small intestine. Hartmann’s procedure was performed for severe anorectal lesions. However, if the lesions worsened, for example, by the development of continuous purulent discharge from multiple anal fistulae, continuous mucous discharge from the remnant colorectum, or anal pain, proctectomy was performed.

We performed regular follow-up examinations every 2 weeks, for up to 3 months after FD at our outpatient center. The follow-up period was measured as the time from FD to the most recent clinical follow-up examination or death. Follow-up examinations were performed until December 31, 2020.

### Outcomes

Outcomes included the incidence of proctectomy in patients with CD after FD, the analysis of uncontrolled Crohn’s anorectal lesions after FD, the restoration of bowel continuity, the cumulative proctectomy rate, and indications for proctectomy (including cancer). Possible risk factors for proctectomy were analyzed to identify significant predictors.

### Statistical analysis

Continuous variables were compared using the Mann–Whitney *U* test. Each factor with a significant P value in a univariate analysis was entered into a stepwise logistic regression model. The data are presented as the median and range. *p* values of < 0.05 were considered to indicate statistical significance. Hazard ratios (HRs) and 95% confidence intervals (CIs) were calculated for all variables in the univariate analyses. The proctectomy rate after fecal diversion was estimated using the Kaplan–Meier method. All statistical analyses were performed using the R software program (version 4.0.2 2020, R Foundation for Statistical Computing, Vienna, Austria).

## Results

### Patients’ characteristics

In total, 174/1218 (14.2%) patients underwent FD because of refractory CD-associated anorectal lesions, defined as actively persistent or symptomatic lesions, wherein optimal medical treatment with surgical drainage had failed. The median observation period from the initial FD was 144 (20–358) months. The baseline characteristics of the 174 patients are presented in Table 1.

**Table 1:** CD patient characteristics after fecal diversion

	Overall	Proctectomy (+)	Proctectomy (−)	*p* *value*
	*n* = 174	*n* = 71	*n* = 103	
Sex (male/female)	118/56	46/25	72/31	0.74
Onset age of CD (years)	19 (4–62)	19 (4–38)	19 (7–62)	0.15
Extent of CD (ileocolitis/colitis/ileitis)	144/30/0	55/16/0	89/14/0	0.1
Rectal involvement	58	30 (42%)	28 (27%)	< 0.01*
Genital fistula	39	15 (21%)	24 (23%)	0.95
Perianal fistula with stricture	91	43 (60%)	48 (46%)	0.02*
Local surgery before fecal diversion	107	48 (67%)	59 (57%)	0.12
Biologics administration before fecal diversion	39 (22%)	17 (24%)	22 (21%)	0.68
Immuno-modulator before fecal diversion	17 (10%)	10 (1%)	7 (0.7%)	0.11
Site of initial stoma (ileostomy/colostomy)	82/92	28 /43	54/49	0.4
Type of stoma at the end of observation (loop stoma/end stoma)	49/125	34/36	15/89	< 0.01*
Duration from diagnosis of CD to fecal diversion (month)	142 (4–358)	161 (20–350)	134 (4–358)	< 0.01*
Biologics administration after fecal diversion	56 (32%)	14 (19%)	42 (40%)	< 0.01*
Immuno-modulator after fecal diversion	24 (14%)	9 (13%)	15 (15%)	0.72
Duration from fecal diversion to proctectomy (months)		59 (9–447)		
Observation time from fecal diversion (month)	144 (20–358)	136 (20–451)	117 (24–323)	0.07

Data are presented as the number (percentage), unless otherwise indicated. Continuous variables are presented as the median (range)

*CD* Crohn’s disease

All 174 patients underwent open laparotomy during the initial FD without proctectomy. One hundred eighteen of the patients were men, and the median onset age of CD was 19 (range, 4–62) years. In total, 144 patients (82.7%) presented with ileocolic compromise, while 30 patients (17.3%) had colitis. Regarding anorectal lesions (there was some overlap), the indications for FD were as follows: complex perianal fistulae (144 patients), anorectal stricture (109 patients), rectal involvement (58 patients), and genital fistula (39 patients). Ninety-one patients had complicating complex perianal fistula and stricture. In total, 107 patients (61.4%) underwent abscess drainage or seton placement before FD. Regarding the initial site of the stoma, ileostomy and colostomy were performed in 82 (47.1%) and 92 (52.9%) patients, respectively. Regarding the type of initial stoma, loop stoma was performed in 33 patients (18.9%), while the rest underwent Hartmann’s procedure. The median duration from the diagnosis of CD to FD was 142 (range, 4–358) months. Sixty-one (35.0%) patients were treated with biologics after FD. Finally, only four patients (2.2%) underwent stoma reversal. Further, 71 patients underwent proctectomy after FD (71/174, 41.3%). The duration of follow-up after FD when proctectomy was performed was 59 (range, 9–447) months. The follow-up period from FD with and without proctectomy was 136 (20–451) months and 117 (24–323) months, respectively.

### Comparison of patients with and without proctectomy after FD

No significant differences in sex, onset of CD, extent of CD, anal lesions (perianal fistula alone, anorectal stricture alone, and genital fistula), stoma site, or duration from the diagnosis of CD to FD were observed between the two groups. The results are summarized in Table 1. However, the patients in the proctectomy group had a significantly higher incidence of rectal involvement and perianal fistula with stricture, higher use of loop stoma/end stoma, longer duration from the diagnosis of CD to FD, and were less frequently treated with biologics after FD, in comparison to the non-proctectomy group (*p* < 0.01, *p* = 0.02, *p* < 0.01, *p* < 0.01, and *p* < 0.01, respectively).

### Risk factors for proctectomy after FD

Univariate and multivariate analyses were performed to identify independent risk factors for conversion to proctectomy after FD, and the results are presented in Table 2. The multivariate logistic regression analysis identified the following independent risk factors for proctectomy after FD: presence of rectal involvement (HR 1.80; 95% CI 1.13–3.05; *p* = 0.02), construction of loop-type stoma (HR 2.21; 95% CI 0.36–3.60; *p* < 0.01), and no administration of biologics after FD (HR 1.92 95% CI 1.05–3.54; *p* = 0.03) (Table 2).

**Table 2:** Logistic regression analysis of the risk factors for transition to proctectomy

Factors	Univariate analysis		Multivariate analysis	
	HR (95% CI)	*p value*	HR (95% CI)	*p value*
Male sex	1.08 (0.66–1.77)	0.74		
Type of CD (ileocolic)	1.62 (0.91–2.90)	0.1		
Rectal involvement	1.91 (1.17–3.12)	0.01*	1.80 (1.13–3.05)	0.02*
Genital fistula	1.01 (0.58–1.77)	0.95		
Perianal fistula and stricture	1.73 (1.06–2.81)	0.02*	1.36 (0.82–2.25)	0.22
Local surgery	1.48 (0.89–2.45)	0.12		
Loop stoma	2.62 (1.63–4.21)	< 0.01*	2.21 (1.36–3.60)	< 0.01*
Ileostomy	1.22 (0.75–1.98)	0.4		
No biologics administration after fecal diversion	2.56 (1.44–4.55)	< 0.01*	1.92 (1.05–3.54)	0.03*

*CI* confidence interval, *CD* Crohn’s disease

### Analysis of uncontrolled Crohn’s anorectal lesion after FD

Uncontrolled Crohn’s anorectal lesion was defined as unimproved symptoms. In total, 129 (74%) of 174 patients showed improved symptoms. The other 45 patients were classified into the proctectomy and non-proctectomy groups (31 and 14 patients, respectively), and their data were compared. The results are summarized in Table 3. Patients in the proctectomy group had a significantly higher incidence of loop stoma/end than those in the non-proctectomy group (*p* = 0.01).

**Table 3:** Uncontrolled Crohn’s anorectal lesion after fecal diversion

	Overall	Proctectomy (+)	Proctectomy (−)	*p value*
	*n* = 45	*n* = 31	*n* = 14	
Sex (male/female)	27/18	20/11	7/7	0.36
Onset age of CD (years)	19 (4–38)	20 (4–38)	19 (5–34)	0.82
Extent of CD (Ileocolitis/Colitis/Ileitis)	33/12/0	22/9/0	11/3/0	0.59
Rectal involvement	15	12 (39%)	3(21%)	0.26
Genital fistula	14	7 (23%)	7 (50%)	0.95
Perianal fistula with stricture	25	18 (58%)	7 (50%)	0.61
Local surgery before fecal diversion	29	21 (68%)	8(57%)	0.49
Site of initial stoma (ileostomy/colostomy)	26/20	16/15	9/5/2022	0.32
Type of stoma at the end of observation (loop stoma/end stoma)	19/26	17/14	2/12	0.01*
Duration from diagnosis of CD to fecal diversion (month)	166 (19–350)	177 (20–350)	114 (19–285)	0.42
Biologics administration after fecal diversion	10 (22%)	6 (19%)	4 (29%)	0.49
Duration from fecal diversion to proctectomy (months)	47(9–447)	48 (10–447)	32 (9–260)	0.88

Data are presented as the number (percentage), unless otherwise indicated. Continuous variables are presented as the median (range)

*CD* Crohn’s disease

### Restoration of bowel continuity after FD, cumulative conversion rate, and indications for proctectomy

**Fig. 1:**
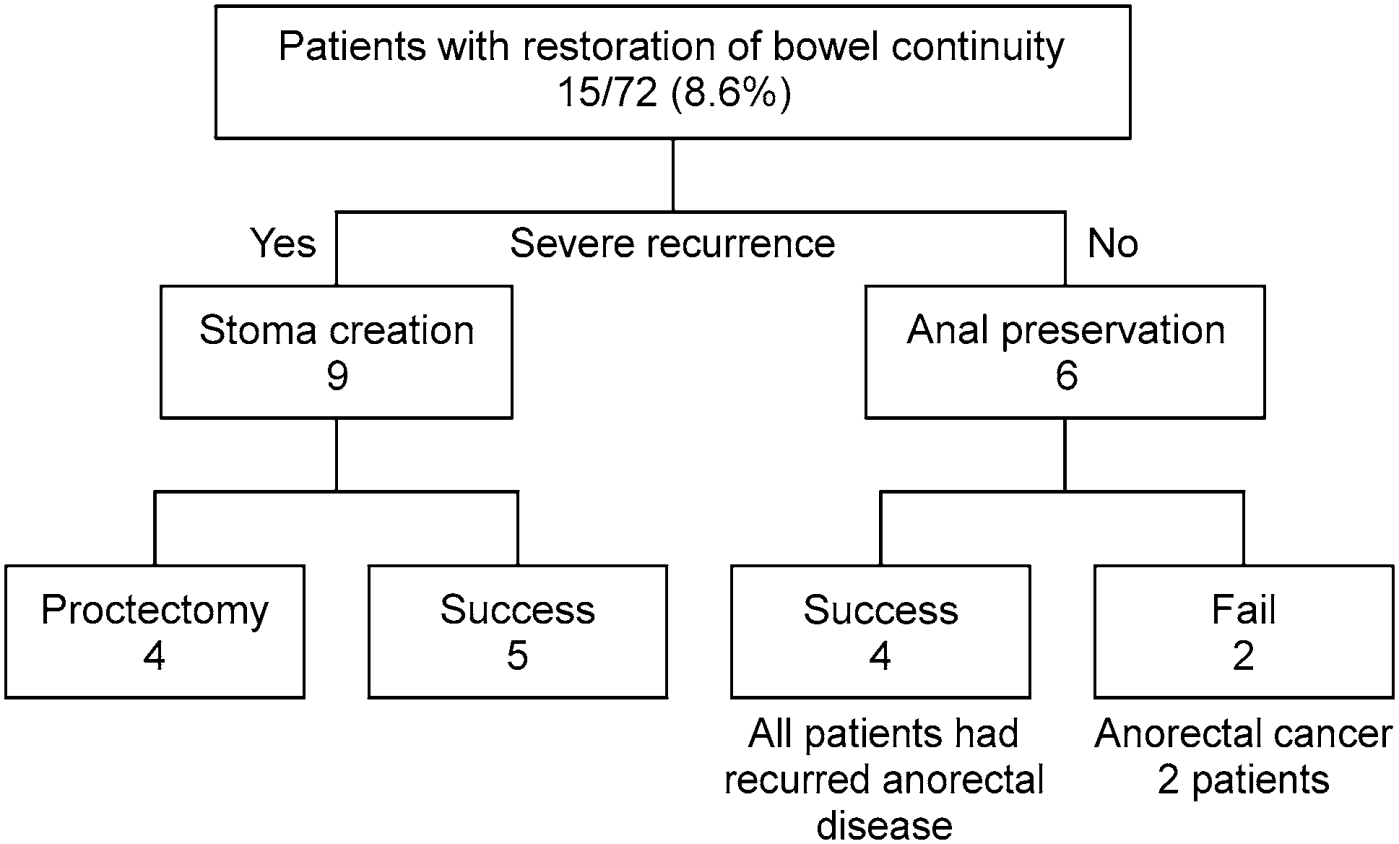
Patients with restoration of bowel continuity

Stoma reversal was performed in 15 of 174 patients (8.6%) (Fig. 1), of which nine patients required re-FD because of an anorectal exacerbation. Four of the nine patients underwent proctectomy, while six underwent anal preservation surgery. Four of the six patients showed restoration of bowel continuity, although they had an anorectal recurrence, while the other two patients who had advanced anorectal cancer died.

**Fig. 2:**
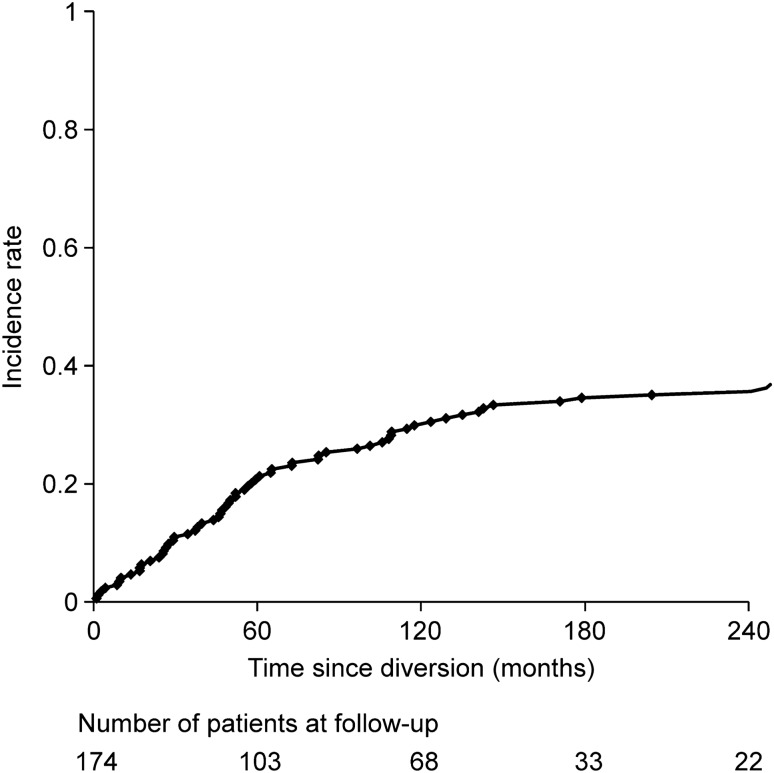
Cumulative conversion rate for proctectomy after fecal diversion

The rate of 10-year cumulative proctectomy after FD was 36.7% (Fig. 2). Table 4 shows the indications for proctectomy after FD. The most common indication for proctectomy (47/72 patients) was refractory anorectal disease (65.2%), while the second most common indication was cancer arising from the diverted colon or rectum in 13/72 (18.0%) patients. Complications related to the diverted colon and rectum, including fistulae between the rectum (stump) and functional intestine, active inflammation, and rectal dilatation due to anorectal stricture, were the indications for proctectomy in 10/72 patients (13.8%). Two patients (2.7%) underwent proctectomy for cancer prevention: one had mucus discharge from the residual rectum, and one developed no symptoms after surgery. Overall, there were 17 cancer patients, including two patients with restoration of bowel continuity and two with FD, in whom proctectomy was not possible because of the tumor size and invasion of other organs. Among these patients, 15 died (15/17, 88%) and two survived; however, one patient experienced pelvic recurrence, and the other developed lung metastasis.

**Table 4:** Indications for proctectomy (*n* = 72)

Indication	Cases
Severe anorectal CD	47
Cancer arising from diverted colon and rectum	13
Fistula between diverted rectum and oral-sided intestine	6
Active inflammation of the diverted colon and rectum	2
Rectal dilatation caused by anorectal stricture	2
Prevention of cancer	2

*CD* Crohn's disease

## Discussion

In this study, we reported that the incidence rate of FD for refractory CD-associated anorectal lesions was 14.2%. A meta-analysis by Singh et al. reported that clinical symptoms improved in two-thirds of patients after FD [8]; similarly, 75% of our patients had an early clinical response. We believe that FD is a relatively effective procedure for treating CD-associated anorectal lesions in terms of symptom relief; however, a few patients may later require proctectomy with a permanent stoma. Therefore, we aimed to determine the proctectomy rate after FD in our center. Several reports have described the proctectomy rates after FD [10–20]. Generally, the proctectomy rate with or without previous FD, ranges from 19.2% to 28.8% [20–24]. The proctectomy rate after FD in our study was 41.3%, which was higher in comparison to previous reports (25%) [13].

In our study, rectal fistula, loop stoma, and non-administration of biologics were identified as independent risk factors for proctectomy after FD. Further, several authors reported that rectal involvement by CD was an independent predictor of proctectomy after FD [13, 20]. These results suggest that rectal involvement may not improve after FD, which was the reason why primary TPC or early completion proctectomy could be considered for this condition [25]. However, no studies have reported loop-type stoma as a risk factor for proctectomy after FD. We speculated that stool flow into the anal side-limb of the loop-type stoma could cause exacerbations of anorectal lesions in our patients. The administration of biologic agents is used to treat patients who do not demonstrate symptomatic improvement after FD. Several studies have reported that biologics were effective for treating perianal disease [20, 26–28]; however, they were not effective for reducing the proctectomy rate. Further, regarding FD, biologic agents were not associated with an increased rate of successful restoration of bowel continuity [10, 11, 20, 29]. Our multivariate analysis demonstrated a lower incidence of proctectomy in patients receiving biologics after FD. There are no reports regarding the proctectomy rate of FD before or after the administration of biologics. We speculate that biologics suppress cancer development due to intestinal remission. However, it is necessary to analyze the effects of biologics after FD in a larger population.

Furthermore, regardless of FD, studies have reported that female sex, duration of CD, history of perineal CD, smoking, and the administration of thiopurines are risk factors for proctectomy [22, 24]. We believe that these risk factors require further investigation. The management of uncontrolled Crohn’s anorectal lesions was controversial. In our study, loop stoma was a risk factor for proctectomy. To our knowledge, there are no other reports indicating loop stoma as a risk factor for proctectomy. However, the exact reason could not be clearly stated. Therefore, it is necessary to further examine this issue in a larger population.

Moreover, many reports have discussed stoma reversal after FD [10–14, 20]. In our study, only 15 patients (8.6%) underwent the restoration of bowel continuity, and it was successful in only four patients (2.3%) who ultimately developed recurrence of anorectal lesions. Hain et al. stated that the rate of stoma reversal was 51%, and that the rate of proctectomy was 26% because of the administration of biologics [20]. We did not include the rate of treatment with biologics in this study, and we may introduce biologics actively and consider stoma reversal in future studies. Two patients with restored bowel continuity developed advanced anorectal cancer and died without undergoing surgery. These patients had severe anorectal symptoms, including anal pain and discharge after stoma reversal. Hence, we should pay close attention to the development of cancer in patients with diversion of the colon and rectum, even after performing stoma reversal. In our study, we performed prophylactic proctectomy in two patients. If patients opt for proctectomy, we need to consider it.

The present study was associated with some limitations. First, this was a retrospective study conducted at a single center. Second, the ethnicity of all patients was Japanese, and differences among races and ethnicities in the occurrence of anorectal lesions may exist. In fact, Japanese patients with CD have a higher frequency of anorectal cancer than European or American patients [30]. Third, the criteria employed for the restoration of bowel continuity were ambiguous.

In conclusion, the risk factors for conversion to proctectomy after FD for residual CD-associated anorectal lesions included the presence of rectal involvement, construction of loop-type stoma, and the absence of treatment with biologics. Considering the low rate of restoration of bowel continuity, proctectomy without previous FD may be an alternative in patients with rectal involvement. Similarly, biologics may decrease the proctectomy rate after FD failure.

## Data Availability

The data underlying this article are available from the corresponding author upon reasonable request.
